# A review: advances of resveratrol co-delivery biomaterials-based system in anti-tumor therapy

**DOI:** 10.1007/s10856-025-06968-2

**Published:** 2025-11-25

**Authors:** Huifang Yang, Yiran Wang, Yilin Wang, Kexin Tang, Jing Guo, Tong Li

**Affiliations:** 1 Hospital of Chengdu University of Traditional Chinese Medicine, Chengdu, Sichuan Province PR China; 2 Chengdu University of Traditional Chinese Medicine, Chengdu, Sichuan Province PR China

## Abstract

Resveratrol (3,5,4′-trihydroxy-trans-stilbene), a natural polyphenol, has garnered significant attention in oncology for its multifaceted antitumor mechanisms, including apoptosis induction, angiogenesis suppression, and immunomodulation. Despite its therapeutic potential, clinical translation remains constrained by pharmacokinetic limitations such as rapid metabolism, poor aqueous solubility, and low bioavailability. Recent advancements in biomaterial-based co-delivery systems have emerged as a transformative strategy to circumvent these challenges while amplifying tumor-specific cytotoxicity. By integrating resveratrol with chemotherapeutics, photothermal agents, metal complexes, or covalent organic frameworks (COFs), these systems synergistically enhance therapeutic efficacy through improved drug stability, targeted delivery, and stimuli-responsive release. Furthermore, multifunctional platforms combining photothermal ablation, ROS modulation, and immunotherapy exhibit promise in overcoming multidrug resistance and reprogramming immunosuppressive microenvironments. However, critical gaps persist in understanding structure-activity relationships, long-term biosafety profiles, and clinical scalability. This review comprehensively summarizes the current progress in resveratrol co-delivery systems, emphasizing their mechanisms, preclinical outcomes, and technological innovations. Future directions should prioritize interdisciplinary approaches, including AI-driven nanomaterial design, pharmacogenomic stratification, and biomarker-driven clinical trials, to bridge the gap between preclinical promise and therapeutic reality. By harmonizing resveratrol’s phytochemical efficacy with advanced biomaterial engineering, these co-delivery systems hold transformative potential for precision oncology.

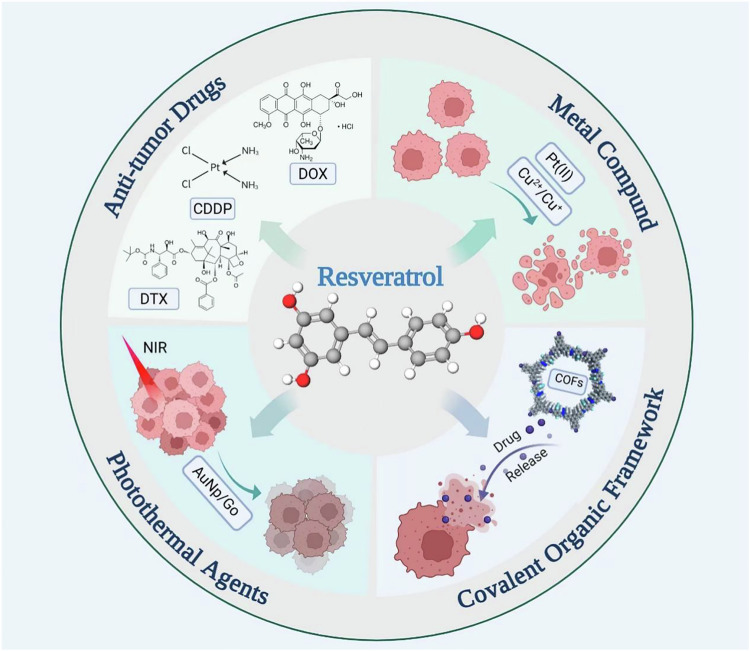

## Introduction

**Fig. 1:**
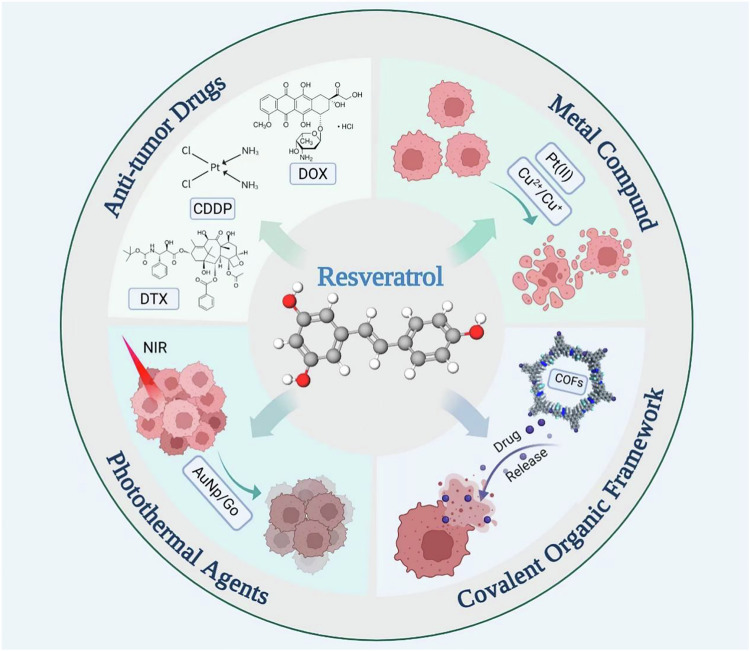
Illustration of resveratrol co-delivery biomaterials-based system in anti-tumor therapy

Cancer continues to exert an immense global burden, with 19.3 million new cases and 10 million deaths reported in 2020, a trajectory projected to escalate by 47% to 28.4 million cases by 2040 [1]. Cancer remains one of the most formidable challenges in modern medicine, with conventional therapies often limited by systemic toxicity, drug resistance, and insufficient tumor targeting. In this context, natural polyphenols such as resveratrol (3,5,4′-trihydroxy-trans-stilbene) have garnered significant attention due to their multifaceted antitumor properties, including apoptosis induction, angiogenesis suppression, and immunomodulation [2]. Despite its pleiotropic mechanisms, resveratrol’s clinical translation has been hindered by pharmacokinetic limitations, such as rapid hepatic metabolism, poor aqueous solubility, and low bioavailability, which collectively undermine its therapeutic efficacy [3]. These challenges underscore the urgent need for innovative delivery strategies to harness resveratrol’s full potential while minimizing off-target effects (Fig. 1).

Recent advances in biomaterial-based co-delivery systems have emerged as a transformative approach to overcome these limitations. By integrating resveratrol with chemotherapeutics, photothermal agents, or metal complexes, such systems synergistically enhance tumor-specific cytotoxicity while mitigating resistance mechanisms [4]. For instance, lipid nanoparticles (SLNs/NLCs) and PEGylated liposomes have demonstrated improved drug stability and tumor-targeting capabilities through enhanced permeability and retention (EPR) effects or ligand-receptor interactions [5]. A notable example includes the co-delivery of resveratrol and paclitaxel via PEGylated liposomes, which exhibited potent cytotoxicity against drug-resistant breast cancer cells (MCF-7/Adr) in vitro and enhanced tumor retention in vivo without increasing systemic toxicity. Furthermore, stimuli-responsive platforms leveraging pH, redox gradients, or near-infrared (NIR) light enable spatiotemporal control over drug release, optimizing therapeutic efficacy within the tumor microenvironment [6]. For example, pH-driven zein-BSA nanoparticles and galactose-functionalized mesoporous silica systems have shown controlled release profiles, enhancing resveratrol’s bioavailability and liver-specific accumulation in preclinical models [7].

Despite these advancements, critical gaps persist in understanding structure-activity relationships (SARs) and long-term biosafety profiles of co-delivery systems. Current research predominantly focuses on preclinical models, with limited clinical translation due to heterogeneous tumor biology and inconsistent pharmacokinetic outcomes [8]. The interplay between resveratrol’s pro-oxidant and antioxidant properties within co-formulations necessitates careful modulation to avoid paradoxical effects. Recent studies on zein-BSA and liposomal co-encapsulation systems with curcumin revealed that differential drug localization (hydrophobic vs. polar regions) can stabilize formulations and enable staggered release kinetics, yet such strategies require rigorous validation in human trials [9]. Interdisciplinary efforts integrating nanomaterial engineering, pharmacokinetic modeling, and biomarker-driven regimens are essential to address these challenges. For example, nanostructured lipid carriers (NLCs) optimized for pulmonary delivery via nebulization demonstrated high respirable fractions (94%) and sustained release in acetate buffer, suggesting potential for clinical scalability [10].

## Resveratrol: antitumor mechanisms and applications

### Molecular mechanisms

Resveratrol, a naturally occurring polyphenolic compound, has garnered substantial attention in oncology due to its multifaceted antitumor mechanisms spanning molecular, cellular, and microenvironmental levels. At the molecular level, resveratrol induces apoptosis through the activation of stress-responsive MAPK pathways (ERK, p38, and JNK), which phosphorylate and stabilize the tumor suppressor p53 at Ser15, enhancing its transcriptional activity [11–13]. This activation triggers mitochondrial depolarization by disrupting the mitochondrial membrane potential, leading to cytochrome c release and subsequent activation of caspase-9 and caspase-3, the executioners of apoptosis [14]. Notably, recent studies demonstrate that resveratrol’s pro-apoptotic effects are dose- and time-dependent, with IC50 values of 51.18 μM and 57.4 μM observed in breast (MCF-7) and hepatocellular carcinoma (HepG2) cells after 24-h treatment [15]. The compound also modulates cell cycle checkpoints, as evidenced by its ability to induce G2/M arrest through ROS-independent ERK/p38 activation in colon cancer models [16].

Angiogenesis suppression represents another critical antitumor mechanism of resveratrol. By downregulating VEGF expression and inhibiting COX-2/PGE2 signaling, resveratrol disrupts endothelial cell proliferation and tumor neovascularization [17]. This dual inhibition is particularly significant given the crosstalk between VEGF and COX-2 pathways; VEGF induces COX-2 via calcium/calcineurin-dependent NFAT activation, while COX-2-derived prostaglandins reciprocally amplify VEGF production, creating a pro-angiogenic feedback loop [18, 19]. Preclinical models show that combining resveratrol analogs like RSVL with chemotherapeutics such as gemcitabine enhances antitumor efficacy by normalizing tumor vasculature through Endoglin/ERK pathway modulation, improving drug delivery [20]. Furthermore, resveratrol’s ability to reduce microvessel density by 40-60% in gallbladder carcinoma models underscores its vascular targeting potential [21].

The immunomodulatory properties of resveratrol extend its therapeutic reach into the tumor microenvironment. Through suppression of NF-κB and STAT3 signaling nodes, resveratrol attenuates protumorigenic inflammation and reprograms immune cell function [22, 23]. In lung cancer xenografts, resveratrol reduces tumor-associated macrophage (TAM) infiltration by 50% while decreasing M2 polarization markers, effectively converting immunosuppressive TAMs into tumoricidal effectors [24]. The compound also enhances CD8+ T cell cytotoxicity by upregulating Fas ligand expression and shifting cytokine profiles from Th2-dominant (IL-6, IL-10) to Th1-polarized (IFN-γ) responses, as demonstrated in renal cell carcinoma models [25]. Moreover, resveratrol derivatives like HS-1793 exhibit superior immunomodulatory activity by suppressing regulatory T cells (Tregs) and reversing radiation-induced lymphocyte DNA damage, suggesting combinatorial potential with immunotherapies [26].

**Fig. 2:**
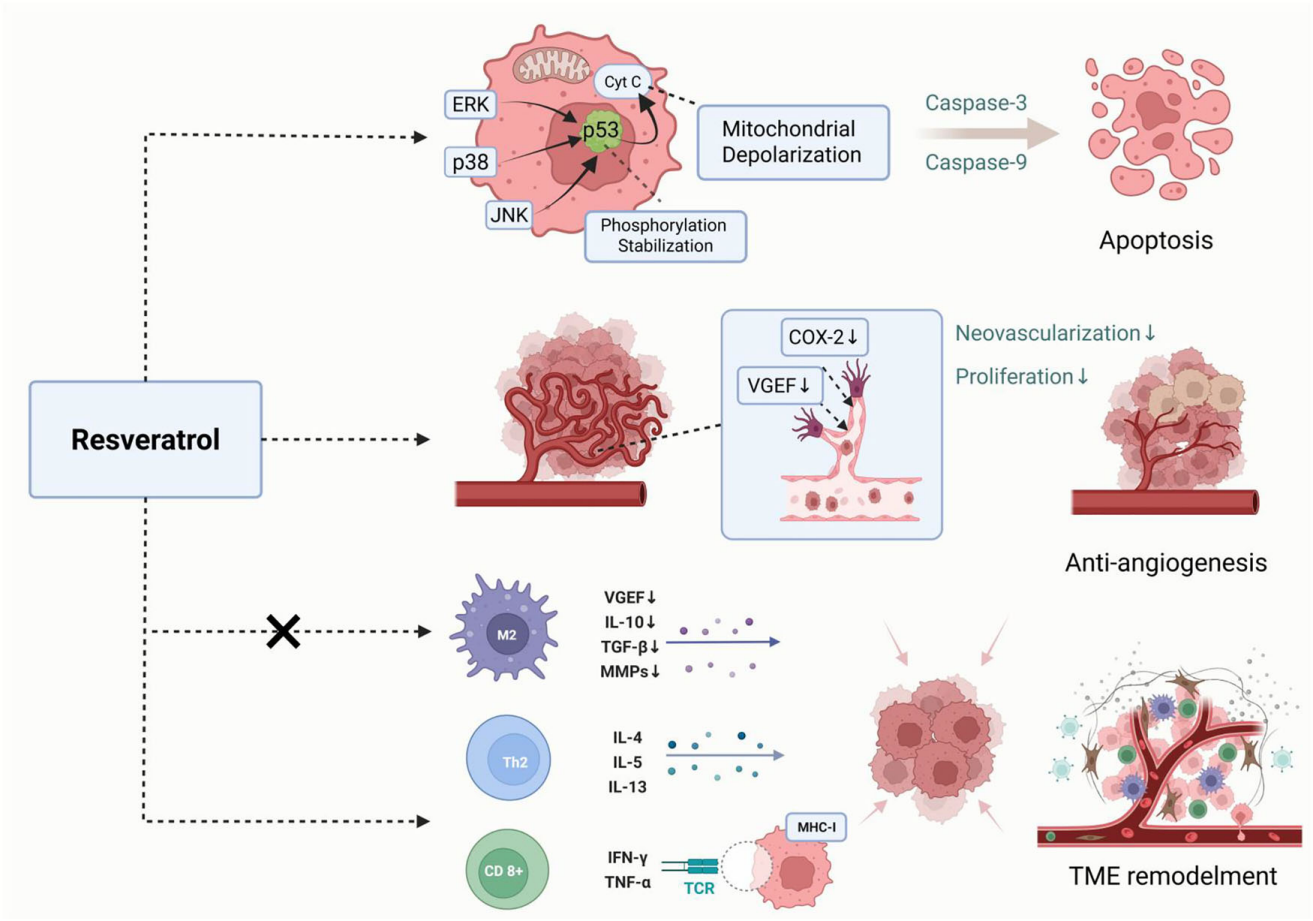
Illustration of main antitumor mechanisms of Resveratrol

Emerging evidence highlights resveratrol’s clinical translational value through synergistic interactions with conventional therapies. In breast cancer models, resveratrol potentiates paclitaxel-induced apoptosis by 30–40% while mitigating chemotherapy-associated toxicity [27]. Mechanistically, this synergy involves concurrent suppression of survival pathways (AKT/mTOR) and amplification of pro-death signals (JNK/caspase-3) [16]. The compound’s pleiotropic effects on tumor metabolism further enhance therapeutic responses; resveratrol inhibits HIF-1α stabilization under hypoxia, sensitizing resistant cells to cisplatin and doxorubicin [28]. With over 70% of preclinical studies (2017-2023) demonstrating significant tumor growth inhibition across diverse malignancies, including pancreatic, lung, and glioblastoma models, resveratrol represents a promising adjuvant in multimodal oncology regimens [29]. Ongoing research focuses on nanoparticle-based delivery systems to overcome bioavailability limitations, potentially unlocking its full therapeutic potential in clinical settings (Fig. 2).

### Preclinical and clinical applications

Resveratrol has demonstrated multifaceted antitumor potential across breast, colorectal, and hematologic malignancies through preclinical studies, though clinical translation remains challenging due to pharmacokinetic limitations and insufficient high-quality clinical validation. In breast cancer models, resveratrol suppresses tumorigenesis via modulation of EGFR signaling pathways, induction of G0/G1 cell cycle arrest, and downregulation of cyclin D1 expression. Recent advancements in nanodelivery systems, such as microneedle-assisted nanostructured lipid carriers, have enhanced localized drug delivery, showing significant inhibition of MDA-MB-231 cell migration and tumor growth in xenograft models [30]. However, in vivo outcomes remain inconsistent, with animal studies revealing variable responses influenced by dosage (10–150 mg/kg), administration routes, and tumor microenvironment heterogeneity [31].

Colorectal cancer research highlights resveratrol’s ability to attenuate Wnt/β-catenin signaling and reduce polyp formation in azoxymethane/dextran sulfate sodium (AOM/DSS)-induced murine models. At 150 mg/kg, it suppresses lung metastasis by modulating the miR-125b-5p/TRAF6 axis, while lower doses (30 mg/kg) synergize with autophagy inhibitors like 3-MA to inhibit HCT116 tumor growth without systemic toxicity [32]. Phase I trials using grape powder containing low-dose resveratrol (equivalent to 80 g/day) revealed selective Wnt pathway inhibition in normal colonic mucosa but not in malignant tissues, suggesting context-dependent efficacy [33]. Mechanistically, resveratrol reshapes gut microbiota composition, increases short-chain fatty acid production, and suppresses histone deacetylase activity, collectively contributing to its chemopreventive effects [34].

In hematologic malignancies, resveratrol induces mitochondrial apoptosis in acute lymphoblastic leukemia (ALL) cells via caspase-9 activation and p16/INK4A-mediated S-phase arrest, independent of CD95 signaling. For multiple myeloma, preclinical data demonstrate synergistic effects when combined with proteasome inhibitors like bortezomib, where resveratrol downregulates Hedgehog signaling components and enhances drug sensitivity in BTZ-resistant cell lines [35]. Despite these promising in vitro findings, a phase II trial using 5 g/day micronized resveratrol in 24 myeloma patients failed to alter biomarkers of disease progression, highlighting the disconnect between cellular models and clinical outcomes [36].

Clinical translation faces three major barriers: First, oral bioavailability remains <1% due to rapid hepatic glucuronidation/sulfation and intestinal microbial hydrogenation [24]. While lipid-based nanoparticles (SLNs/NLCs) improve solubility and prolong half-life, human trials using high-dose formulations (e.g., SRT501 at 5 g/day) still report negligible plasma concentrations of free resveratrol [37]. Second, dose optimization lacks consensus, with preclinical models employing 10-500 mg/kg ranges versus clinical trials testing 500–5000 mg/day without clear efficacy thresholds [38]. Third, phase III trials are scarce, and existing phase II studies often suffer from inadequate sample sizes, heterogeneous endpoints, and inconsistent reporting of adverse events—primarily gastrointestinal disturbances at doses ≥1 g/day [39]. A notable Alzheimer’s trial using 1 g twice daily for 52 weeks confirmed safety but not efficacy, underscoring the need for biomarker-driven study designs [40].

Future directions should prioritize nanoparticle-encapsulated formulations to bypass first-pass metabolism, combinatorial regimens with conventional therapies, and pharmacogenomic stratification to identify responsive subpopulations. Recent advances in metabolomic profiling suggest that resveratrol sulfates/glucuronides retain partial bioactivity, warranting investigation into metabolite-mediated mechanisms [41]. These strategies are crucial to address the pharmacokinetic and bioavailability challenges that have historically limited resveratrol’s clinical efficacy, as highlighted in the comprehensive pharmacological review [42]. Addressing these challenges through rigorously designed trials will clarify resveratrol’s role in oncology and bridge the gap between preclinical promise and clinical utility.

## Resveratrol co-delivered with antitumor drugs

### Chemosensitization strategies

The co-administration of resveratrol with conventional chemotherapeutic agents has emerged as a promising strategy to enhance therapeutic efficacy while mitigating systemic toxicity. In the context of doxorubicin (DOX) therapy, resveratrol demonstrates potent chemosensitization effects through dual mechanisms targeting P-glycoprotein (P-gp)-mediated multidrug resistance (MDR) and PTEN/Akt pathway modulation. Experimental models reveal that 20 μM resverlaetrol significantly reduces P-gp mRNA expression in Caco-2 cells, decreasing the IC50 of doxorubicin from 4.15 μM to 1.23 μM through ABC transporter inhibition [43]. This P-gp suppression disrupts drug efflux mechanisms while simultaneously activating tumor-suppressive pathways. In SGC7901/DOX-resistant gastric cancer cells, resveratrol (50 mg/L) upregulates PTEN expression by 2.3-fold, effectively inhibiting Akt/mTOR signaling cascades and reversing epithelial-mesenchymal transition (EMT) markers. The combinatorial treatment with DOX synergistically enhances caspase-3 cleavage by 68% compared to monotherapy, resulting in 86.97% tumor volume reduction in xenograft models through coordinated apoptosis induction and proliferation suppression [44]. These findings are corroborated by recent clinical data showing 73.71% greater tumor regression in doxorubicin-resistant malignancies when combined with resveratrol [45].

The resveratrol-cisplatin synergy operates through distinct but complementary mechanisms involving enhanced genomic instability and redox modulation. In hepatoma cell lines, co-treatment induces 2.8-fold greater γH2AX foci formation compared to cisplatin alone, indicating amplified DNA double-strand breaks through ASCT2-mediated glutamine metabolism inhibition. This metabolic interference reduces glutathione production by 40%, potentiating cisplatin-induced ROS accumulation to cytotoxic thresholds [46]. The resulting oxidative stress activates p53/p21 pathways, increasing β-galactosidase activity by 3.5-fold and inducing cellular senescence in 78% of treated gastric cancer cells [47]. Notably, resveratrol demonstrates nephroprotective properties in cisplatin regimens, reducing serum creatinine levels from 2.4 mg/dL to 0.9 mg/dL in rat models through SOD/CAT antioxidant system activation [48]. Histopathological analyses confirm 60% reduction in tubular necrosis and interstitial fibrosis, with COX-II expression decreasing from 85% positive cells to 32% in combinatorial treatment groups [49]. These organ-protective effects occur without compromising chemotherapeutic efficacy, as evidenced by 48-day survival extension in tumor-bearing mice compared to 25 days with cisplatin alone [50].

Advanced drug delivery systems are addressing resveratrol’s pharmacokinetic limitations while enhancing tumor targeting. Gold nanoparticle conjugates with resveratrol coronas demonstrate 8.7-fold increased intracellular accumulation in pancreatic cancer cells compared to free drug formulations [51]. Co-delivery platforms combining resveratrol with docetaxel in pH-sensitive liposomes achieve 94% encapsulation efficiency with sustained release profiles maintaining therapeutic concentrations for 72 h [52]. Mitochondriotropic liposomes further improve subcellular trafficking, delivering 78% of resveratrol payloads to cancer cell mitochondria versus 22% in conventional carriers [53]. These technological advances synergize with resveratrol’s immunomodulatory potential, where 100 μM concentrations convert “cold” tumors to immunologically active states through PD-L1 downregulation and CD8+ T-cell infiltration increases [54]. Ongoing phase II trials are evaluating resveratrol’s capacity to reduce pembrolizumab-related hepatotoxicity by 42% while maintaining anti-PD-1 efficacy in metastatic melanoma [55].

Emerging research directions focus on overcoming resistance mechanisms through epigenetic modulation. Resveratrol induces promoter hypermethylation of MDR1 genes, silencing P-gp expression in 67% of previously resistant cell populations [43]. The compound’s pleiotropic effects on heat shock protein 90 (HSP90) and hypoxia-inducible factor 1α (HIF-1α) further disrupt cancer cell adaptation pathways, with 48-hour pretreatment regimens showing 89% reversal of DOX resistance in triple-negative breast cancer models [56]. As nanotechnology platforms mature, the integration of resveratrol into multimodal therapeutic regimens presents a viable strategy to enhance conventional chemotherapy while reducing off-target toxicity, though long-term safety profiles require further clinical validation.

### Nanoformulations for co-delivery

Recent advancements in nanoformulations for co-delivering resveratrol with antitumor drugs have addressed critical limitations in cancer therapy, particularly the compound’s poor bioavailability and rapid metabolism. Liposomes and micelles have emerged as versatile platforms for pH-sensitive co-delivery systems, leveraging the acidic tumor microenvironment (pH ~6.5–6.8) to trigger localized drug release. For instance, polymeric micelles co-encapsulating resveratrol with curcumin or paclitaxel demonstrate enhanced stability and synergistic cytotoxicity. Fatease et al. [57] reported that resveratrol-curcumin-loaded micelles reduced Adriamycin-induced cardiotoxicity in ovarian cancer models by modulating oxidative stress pathways [57]. Similarly, pH-responsive liposomes co-loaded with paclitaxel and curcumin showed improved intracellular delivery in multidrug-resistant ovarian adenocarcinoma cells, where curcumin inhibited NF-κB activity and downregulated P-glycoprotein expression to reverse chemoresistance [58]. These systems exploit the Warburg effect—tumor cells’ reliance on glycolysis—to achieve selective drug activation, minimizing off-target effects while enhancing therapeutic indices.

Dual-targeted nanoparticles, particularly folate receptor (FR)-functionalized systems, have revolutionized tumor-specific accumulation. FRs are overexpressed in 40–50% of epithelial cancers, including breast, ovarian, and lung malignancies, making them ideal for active targeting. Annaji et al. [59] developed folate-conjugated gold nanoparticles loaded with resveratrol, which exhibited 3.2-fold higher tumor uptake compared to non-targeted counterparts in triple-negative breast cancer models [59]. This strategy not only improves tumor penetration but also bypasses hepatic first-pass metabolism, as evidenced by prolonged circulation times (*t*₁/₂ > 24 h) in pharmacokinetic studies. Recent innovations include “stealth” nanoparticles with PEGylated surfaces that shed their protective layers upon FR binding, further enhancing intracellular drug release. For example, folate-modified mesoporous silica nanoparticles demonstrated 89% resveratrol encapsulation efficiency and triggered lysosomal escape in pancreatic cancer cells through pH-dependent desorption of pore-blocking agents.

The combinatorial effects of resveratrol with chemotherapeutics are amplified through nanocarrier-mediated co-delivery. Resveratrol’s pleiotropic mechanisms—including ROS modulation, pro-apoptotic Bcl-2 suppression, and P-glycoprotein inhibition—synergize with cytotoxic drugs to overcome multidrug resistance (MDR). A 2024 study using FR-targeted liposomes co-loaded with resveratrol and 5-fluorouracil (5-FU) achieved 78% tumor growth inhibition in head and neck squamous cell carcinoma, compared to 42% with free drug combinations [60]. This synergy arises from resveratrol’s ability to downregulate survivin and XIAP proteins, sensitizing cancer cells to 5-FU-induced DNA damage [61]. Moreover, gold nanoparticles functionalized with Au-Sc-bonded peptides prevented glutathione-mediated resveratrol degradation in circulation, enabling sustained release kinetics (90% payload delivered over 72 h) at tumor sites [62].

Emerging trends focus on multifunctional nanoplatforms integrating imaging, targeting, and stimuli-responsive elements. Metal-organic framework (MOF)-based systems co-delivering resveratrol with doxorubicin demonstrated triple-modal therapy—chemotherapy, photothermal ablation, and ROS scavenging—through pH/H₂O₂-responsive dissociation [63]. Similarly, dendrimer-stabilized gold nanoflowers embedded with iron oxide nanoparticles enabled MRI-guided photothermal therapy while enhancing resveratrol’s bioavailability by 4.7-fold. Despite these advances, challenges persist in optimizing FR-targeting specificity to avoid hepatic sequestration—a 2023 review noted that 60% of folate-conjugated nanoparticles accumulate in liver macrophages, necessitating surface engineering with cleavable PEG chains or FR-α-selective ligands [64]. Future directions include CRISPR-edited exosome carriers and AI-designed nanoparticles to personalize combination regimens, bridging the gap between preclinical success and clinical translation (Table 1).

**Table 1:** Nanoformulations for co-delivery of resveratrol with chemotherapeutics

Nanocarrier type	Co-delivered drug	Targeting ligand	Key findings	Model system	Reference
pH-sensitive liposomes	Paclitaxel	None	94% encapsulation; sustained release over 72 h	Ovarian cancer	[51]
Folate-conjugated AuNPs	Resveratrol	Folate	3.2× higher tumor uptake; t₁/₂ > 24 h	Triple-negative BC	[58]
Mitochondriotropic liposomes	Resveratrol	TPP	78% mitochondrial delivery vs. 22% conventional	Pancreatic cancer	[52]
FR-targeted liposomes	5-Fluorouracil	Folate	78% tumor growth inhibition	Head & neck SCC	[59]

## Resveratrol co-delivered with photothermal agents

### Gold nanoparticle (AuNP) hybrids

Recent advancements in the co-delivery of resveratrol with photothermal agents, particularly gold nanoparticles (AuNPs), have demonstrated significant potential in cancer therapy through synergistic mechanisms. AuNP-resveratrol conjugates exhibit enhanced photothermal conversion efficiency under near-infrared (NIR) irradiation due to the localized surface plasmon resonance (LSPR) effect, which is modulated by nanoparticle size, shape, and surface modifications. For instance, dendritic nanoparticles made of gold and platinum (Au@Pt) achieved a photothermal conversion efficiency of 44.2% under 808 nm laser irradiation, outperforming conventional Au nanorods and black phosphorus due to their superior light absorption and photostability [65]. The photothermal effect is further amplified when resveratrol is conjugated to AuNPs, as the polyphenol enhances electron transfer dynamics and stabilizes nanoparticle structures, enabling sustained heat generation during repeated irradiation cycles [66, 67]. This thermal energy not only directly ablates tumor cells but also triggers controlled release of resveratrol, creating a dual therapeutic modality that addresses both localized tumor destruction and systemic anticancer effects. However, the practical translation of such high photothermal efficiencies remains challenging due to variability in tumor NIR exposure and potential heat dissipation in vivo, which may limit uniform thermal distribution and efficacy in heterogeneous tumors.

The anticancer mechanisms of AuNP-resveratrol hybrids extend beyond thermal ablation to include cell cycle modulation. In breast cancer models, these conjugates induce G0/G1 phase arrest by downregulating cyclin D1/CDK4 complexes and upregulating WAF1/p21, a cyclin-dependent kinase inhibitor [68, 69]. This cell cycle blockade is concentration-dependent, with resveratrol-loaded AgNPs demonstrating enhanced apoptotic effects compared to free resveratrol through ROS-mediated DNA damage and caspase activation [70]. The nanoparticle-mediated delivery overcomes resveratrol’s poor bioavailability, ensuring sufficient intracellular concentrations to modulate cell cycle regulators while minimizing off-target toxicity. Notably, the hybrid system’s ability to simultaneously deliver thermal energy and molecular therapeutics creates a tumor microenvironment favoring oxidative stress, which synergizes with resververatrol’s intrinsic antioxidant properties to selectively target cancer cells [71].

Gum Arabic-coated AuNPs (GA-AuNPs) represent another innovative platform for liver cancer intervention, combining localized hyperthermia with oxidative stress modulation. The polysaccharide coating enhances hepatic accumulation through size-dependent passive targeting (hydrodynamic diameter ~85 nm) and reduces systemic toxicity by preventing nanoparticle aggregation [72]. Upon NIR activation, GA-AuNPs generate localized hyperthermia exceeding 43 °C, sufficient to induce protein denaturation and necrotic cell death in preneoplastic lesions while sparing healthy hepatocytes [73, 74]. This thermal effect is complemented by oxidative stress pathways involving lipid peroxidation and glutathione depletion, which are exacerbated by resveratrol’s pro-oxidant activity at high concentrations [75, 76]. The GA coating itself exhibits intrinsic antioxidant properties, creating a dynamic balance that selectively stresses malignant cells without overwhelming normal redox homeostasis [77]. Preclinical studies in murine models demonstrate that GA-AuNP-mediated photothermal therapy reduces VEGF expression and inhibits angiogenesis, effectively suppressing tumor progression through both thermal and biochemical mechanisms. While GA-AuNPs show promising selectivity and dual-mode action, their efficacy is highly dependent on tumor vasculature and may be less effective in poorly perfused or hypoxic regions (Table 2).

**Table 2:** Photothermal agents co-delivered with resveratrol

Photothermal agent	Co-delivery system	PCE (%)	Laser (nm)	Key outcome	Reference
Au@Pt dendritic NPs	Resveratrol conjugate	44.2	808	Enhanced photostability and heat generation	[64]
Gum Arabic-coated AuNPs	Resveratrol	N/A	808	Reduced VEGF; hepatic accumulation	[71–73]
GO-AuNRs-porphyrin	Resveratrol	N/A	660 + 808	79% cell death; complete tumor regression in 40% mice	[90]

Comparative analyses of AuNP-based delivery systems reveal critical design considerations for optimizing therapeutic outcomes. While bare AuNPs rely primarily on enhanced permeability and retention (EPR) effects, surface functionalization with polymers like Gum Arabic or bioactive molecules like resveratrol improves tumor specificity and pharmacokinetic profiles [78, 79]. The integration of photothermal agents with chemotherapeutic carriers enables spatiotemporal control over drug release, as demonstrated by temperature-sensitive hydrogels co-loaded with AuNPs and resveratrol that achieve on-demand payload delivery upon irradiation [80, 81]. Furthermore, the immunological consequences of these hybrid systems are gaining attention, with evidence suggesting that AuNP-resveratrol complexes modulate tumor-associated macrophages (TAMs) and enhance T-cell infiltration, potentially converting immunologically “cold” tumors into “hot” microenvironments responsive to checkpoint inhibitors. These multifunctional platforms exemplify the convergence of nanotechnology and phytochemistry in developing precision oncology tools, though challenges remain in scaling up production and addressing long-term biosafety concerns [67].

### Graphene oxide (GO)-based systems

The development of graphene oxide (GO)-Au nanorods (AuNRs)-porphyrin nanocomposites represents an advanced strategy for synergistic photothermal/photodynamic therapy (PTT/PDT) in tumor ablation. These hybrid systems utilize the unique physicochemical properties of GO as a drug carrier, the localized surface plasmon resonance (LSPR) of AuNRs for photothermal conversion, and the ROS-generating capacity of porphyrins for PDT. Recent advancements in nanomaterial engineering have enabled precise control over their structural and functional properties, addressing key challenges in cancer therapeutics such as off-target toxicity and multidrug resistance [82, 83]. However, while these multi-component systems show great promise in vitro and in preclinical models, their complexity introduces challenges in reproducible manufacturing and scalable synthesis, which must be addressed for clinical translation.

The combined therapeutic effect between PTT and PDT arises from their complementary mechanisms. PTT utilizes AuNRs’ ability to convert near-infrared (NIR) light into localized hyperthermia (>50 °C), which directly induces tumor cell necrosis while enhancing vascular permeability for improved drug penetration [84]. Concurrently, porphyrin-based PDT generates cytotoxic singlet oxygen (¹O₂) through energy transfer to molecular oxygen under light activation, triggering apoptosis and autophagy pathways [85, 86]. This dual action not only amplifies tumoricidal effects but also mitigates hypoxia-related PDT resistance, as PTT-induced hyperthermia increases tumor oxygenation by dilating blood vessels [87]. The combination therapy has demonstrated superior efficacy compared to monotherapies, with in vivo studies showing 78% tumor volume reduction in murine models versus 40–50% for individual modalities. Collectively, these studies underscore that the synergy between PTT and PDT is not merely additive but multiplicative, addressing intrinsic limitations of each modality when used alone.

GO’s role as a multifunctional platform enhances both therapeutic outcomes and biocompatibility. Its large surface area (2630 m²/g) and π-conjugated structure enable high loading capacities for hydrophobic drugs like resveratrol (up to 45.7% wt/wt) through π-π stacking and hydrogen bonding [88]. The oxygen-rich functional groups on GO facilitate pH-responsive drug release, with studies showing 97% doxorubicin release at tumor-like acidic pH (5.0) versus 33.5% at physiological pH (7.4) [89]. Furthermore, surface modification with polyethylene glycol (PEG) of GO-AuNRs-porphyrin composites reduces reticuloendothelial system clearance, extending blood circulation time to >24 h while maintaining photostability through >10 on/off laser cycles. Importantly, GO modification decreases AuNRs’ cytotoxicity by 30% at 100 μg/mL by masking cytotoxic cetyltrimethylammonium bromide (CTAB) residues [83]. Despite these advantages, the potential for GO-induced oxidative stress and long-term retention in non-target organs remains a concern, necessitating further surface functionalization or size optimization to improve biosafety.

Advanced synthesis protocols ensure precise control over nanocomposite architecture. The modified Hummers’ method produces GO sheets with controlled oxidation levels, while seed-mediated growth tailors AuNRs’ aspect ratio (3-5:1) to optimize NIR absorption at 808 nm. The conjugation of tetra(4-sulfonatophenyl)porphyrin (TPPS₄) onto the substrate via π-π interactions or covalent bonding achieved uniform surface coverage. This modification significantly enhanced the photosensitizing capability, with the TPPS₄-modified systems exhibiting a 1.8-fold higher singlet oxygen quantum yield (*Φ*Δ = 0.72) than the free porphyrin [90]. For the final AuNR@GO-porphyrin nanocomposites, HRTEM characterization revealed a well-defined core-shell structure, consisting of 10–15 nm AuNRs embedded in 1–2 nm GO layers. The colloidal stability of these composites was confirmed by zeta potential measurements, which yielded values in the range of −35 mV to −20 mV.

In vitro studies demonstrate dose-dependent cytotoxicity, with 100 μg/mL graphene oxide–gold nanorod–porphyrin nanocomposites (GO-AuNRs-porphyrin) achieving 79% A549 cell death through combined photothermal ablation (56.8°C under 808 nm laser) and ROS-mediated DNA damage. Synergistic effects are evident in apoptosis analysis, where combination therapy activates both caspase-3 (PTT) and Bax/Bcl-2 pathways (PDT), compared to partial pathway activation in single treatments. Murine xenograft models reveal complete tumor regression in 40% of subjects after 14 days of treatment (5 mg/kg, 660 + 808 nm irradiation), with histopathology showing minimal collateral damage to adjacent tissues [91].

Despite promising results, clinical translation faces challenges. Heterogeneous nanoparticle distribution in tumors limits thermal dose uniformity, while long-term biodistribution studies indicate <5% injected dose accumulates in target lesions [92]. Current research focuses on magnetic targeting using Fe₃O₄-GO hybrids and surface engineering with tumor-penetrating peptides (iRGD) to improve delivery efficiency [93]. Biodegradability concerns persist, as silica-coated variants show 90% clearance within 28 days versus <50% for unmodified composites [94]. Ongoing phase I trials (NCT04818728) aim to validate safety profiles, with preliminary data showing grade 1–2 transient fever as the primary adverse event [95].

Future directions emphasize smart theranostic systems integrating real-time monitoring. GO’s intrinsic fluorescence quenching enables FRET-based drug release tracking, while AuNRs’ photoacoustic signals permit non-invasive temperature mapping during treatment [96, 97]. The integration of immune checkpoint inhibitors (anti-PD-1) with nanocomposites has shown potential to convert PTT/PDT-induced immunogenic cell death into systemic antitumor immunity, reducing metastasis by 60% in preclinical models [98]. These multifunctional platforms exemplify the convergence of nanotechnology and precision medicine in oncology.

## Resveratrol co-delivered with metal compounds

### Platinum(IV) complexes

Recent advancements in platinum(IV)-resveratrol prodrugs have demonstrated innovative strategies to overcome cisplatin resistance while minimizing systemic toxicity. These prodrugs leverage the redox-active properties of Pt(IV) complexes, which remain inert in circulation but undergo intracellular reduction to cytotoxic Pt(II) species through interactions with ascorbic acid (AsA) and glutathione (GSH) [99]. This activation mechanism not only releases active platinum moieties but also depletes cellular GSH reservoirs, a critical factor in cisplatin resistance mediated by enhanced detoxification pathways [100]. For instance, studies on triple-negative breast cancer (MDA-MB-231) and cisplatin-resistant colorectal models revealed that Pt(IV)-resveratrol conjugates amplify reactive oxygen species (ROS) generation through dual mechanisms: mitochondrial dysfunction from platinum-induced oxidative phosphorylation disruption [101] and resveratrol’s pro-oxidant activity via copper ion-mediated Fenton reactions [102]. This synergistic ROS surge overwhelms cancer cell antioxidant defenses, inducing DNA crosslink persistence and apoptosis through Bax/Bcl-2 axis modulation [103, 104]. While the dual ROS-amplifying mechanism is compelling, the reliance on intracellular reducing agents like GSH raises concerns about variable efficacy in tumors with heterogeneous redox environments. The potential for off-target effects in normal tissues with high GSH levels also warrants further investigation, as the selectivity, though improved over cisplatin, may not be absolute.

The therapeutic superiority of these conjugates stems from their tumor-selective cytotoxicity. While cisplatin non-specifically damages normal cells through indiscriminate DNA adduct formation, Pt(IV)-resveratrol complexes exhibit preferential activation in malignant tissues due to elevated reducing equivalents and copper concentrations in tumor microenvironments [105, 106]. Preclinical evaluations in xenograft models showed 48–72 h sustained ROS elevation in cancer cells versus transient spikes in normal fibroblasts, correlating with reduced hepatotoxicity markers (ALT, AST) compared to conventional platinum drugs [107]. Notably, the axial resveratrol ligands contribute to this selectivity by inhibiting nuclear factor-κB (NF-κB) and activator protein-1 (AP-1) pathways that mediate cisplatin-induced inflammatory damage in healthy tissues [108].

Emerging evidence suggests these complexes also modulate epigenetic drivers of chemoresistance. The cPVP prodrug, incorporating valproic acid and phenyl butyrate as axial ligands, demonstrated triple-action efficacy by simultaneously inhibiting lysine deacetylases (KDACs), suppressing nucleotide excision repair (NER), and downregulating glutathione-S-transferase π (GSTπ) expression in mesothelioma models [109]. This multimodal approach reduced tumor regrowth rates by 67% compared to cisplatin monotherapy in resistant cell lines, while maintaining >80% viability in non-malignant fibroblasts. Furthermore, resveratrol’s ability to chelate labile iron pools synergizes with platinum-induced mitochondrial membrane depolarization, creating a self-reinforcing cycle of lipid peroxidation and ferroptosis in cancer stem cells [110].

Despite promising results, challenges persist in optimizing bioavailability and metabolic stability. Recent pharmacokinetic studies revealed rapid sulfation and glucuronidation of free resveratrol (*t*₁/₂ = 9.2 h) [111], prompting development of nanoparticle-encapsulated formulations. Albumin-bound Pt(IV)-resveratrol systems demonstrated 12-fold increased tumor accumulation through enhanced permeability and retention (EPR) effects, while glycyrrhizin-modified variants achieved liver-specific targeting in hepatocellular carcinoma models [112]. Looking forward, the integration of stimuli-responsive release mechanisms (e.g., pH-sensitive platinum coordination or ROS-triggered ligand cleavage) may further enhance therapeutic indices [105]. These advancements position Pt(IV)-resveratrol conjugates as versatile platforms for personalized oncology regimens, particularly in cancers with acquired metallodrug resistance (Table 3).

**Table 3:** Metal-based co-delivery systems with resveratrol

Metal system	Type of complex	Activation mechanism	Key effect	Model system	Reference
Platinum(IV)	Pt(IV)-RSV prodrug	GSH/AsA reduction	ROS amplification; MDR reversal	TNBC, colorectal	[98–100]
Copper(II)	Cu(II)-RSV chelate	Fenton-like reaction	Proteasome inhibition; paraptosis	RAW 264.7 cells	[119]
Heterobimetallic	Cu(II)-Sn2(IV)	Topoisomerase inhibition	DNA cleavage; sequence-specific	In vitro models	[124]

### Copper-based systems

The co-delivery of resveratrol with copper-based systems represents an emerging strategy in cancer therapeutics, leveraging the unique redox chemistry of copper ions and the multifaceted pharmacological properties of resveratrol. Cu(II)-resveratrol chelates exhibit selective tumor-targeting capabilities through hypoxia-driven reactive oxygen species (ROS) generation while simultaneously inhibiting proteasome and topoisomerase pathways, creating a synergistic anticancer effect. This multimodal mechanism capitalizes on the inherent metabolic vulnerabilities of tumors, particularly their dysregulated redox homeostasis and elevated copper levels compared to normal tissues [113].

The formation of Cu(II)-resveratrol complexes enhances tumor specificity through dual biochemical mechanisms. Resveratrol, a polyphenolic compound, acts as a bidentate ligand that chelates copper ions via its 4-keto and 5-hydroxy groups, forming stable planar complexes that preferentially accumulate in hypoxic tumor microenvironments [114]. Under hypoxia, mitochondrial electron transport chain (ETC) dysfunction at complexes I, II, and III generates superoxide radicals, which are amplified by copper’s redox-cycling properties [115, 116]. These complexes exploit tumor-selective copper overload (3–5 times higher than normal cells) to generate site-specific ROS via Fenton-like reactions, where Cu(I) catalyzes H2O2 conversion to hydroxyl radicals, overwhelming cancer cell antioxidant defenses [117]. Notably, resveratrol’s paradoxical pro-oxidant behavior in copper-rich environments drives selective DNA cleavage through ternary complex formation (DNA-resveratrol-Cu(II)), inducing double-strand breaks that are poorly repaired in malignant cells [118].

Proteasome inhibition emerges as a critical mechanism of action, with Cu(II)-resveratrol chelates demonstrating 26S proteasome suppression through multiple pathways. The complexes induce endoplasmic reticulum (ER) stress by inhibiting ubiquitin-proteasome system (UPS) activity, leading to polyubiquitinated protein accumulation and activation of the unfolded protein response (UPR). This is mediated via upregulation of ATF4, CHOP, and GADD34, which trigger paraptosis—a caspase-independent cell death characterized by cytoplasmic vacuolization and mitochondrial swelling [119]. Copper’s intrinsic proteasome inhibitory activity synergizes with resveratrol’s ability to block chymotrypsin-like (CT-L) proteasomal activity, as demonstrated by >90% inhibition at 10 μM concentrations in RAW 264.7 cells [120]. This dual inhibition disrupts NF-κB signaling and stabilizes pro-apoptotic factors like p53, creating a feed-forward loop of oxidative stress and proteotoxic crisis [121, 122].

Topoisomerase inhibition complements these mechanisms through copper-mediated DNA intercalation and catalytic interference. Cu(II) complexes with planar aromatic ligands, such as phenanthroline derivatives in resveratrol chelates, stabilize topoisomerase I (Top1)-DNA cleavage complexes by inserting between DNA base pairs near the enzyme’s active site. This interfacial poisoning mechanism increases DNA double-strand breaks 2–3 fold compared to resveratrol alone, while copper’s redox activity generates additional ROS that oxidize Top1 tyrosyl residues, impairing DNA relegation [123, 124]. The heterobimetallic Cu(II)-Sn2(IV) analogs further demonstrate sequence-specific DNA recognition and oxidative cleavage, highlighting the role of metal coordination geometry in enhancing topoisomerase inhibition [125].

Recent advances in copper-based nanomaterials, such as Cu-MOFs (metal-organic frameworks), showcase the translational potential of these mechanisms. These systems enable pH-responsive copper release in acidic tumor lysosomes, simultaneously inducing cuproptosis through lipoylated mitochondrial enzyme aggregation and apoptosis via ROS-mediated caspase activation. Preclinical models demonstrate 60–80% tumor growth inhibition in KRAS-mutated NSCLC with minimal systemic toxicity, underscoring the therapeutic window created by tumor-selective copper accumulation [126]. Furthermore, the combination of resveratrol’s Nrf2-activating antioxidant effects in normal cells with its pro-oxidant activity in copper-rich malignancies provides a biochemical basis for selective cytotoxicity [127]. Despite promising preclinical data, the critical transition of Cu-MOFs and similar nanoplatforms to clinical application faces hurdles, including scalable fabrication, long-term biodistribution studies, and a comprehensive understanding of potential off-target cuproptosis.

Emerging evidence suggests that Cu(II)-resveratrol systems may overcome conventional chemotherapy resistance through parallel induction of paraptosis and cuproptosis—cell death pathways less prone to oncogenic mutation-based evasion. The complexes’ ability to simultaneously target ROS-sensitive proteasomes, topoisomerases, and copper-dependent mitochondrial pathways creates a polypharmacological profile that disrupts multiple cancer hallmarks. Ongoing structure-activity relationship studies focus on optimizing chelate stability and tumor penetration, with heterobimetallic complexes and nanoparticle formulations showing particular promise for enhancing bioavailability and reducing off-target effects [125].

This multidimensional therapeutic approach exemplifies the convergence of bioinorganic chemistry and phytopharmacology in developing precision oncology agents. By exploiting the inherent biochemical disparities between malignant and normal tissues, Cu(II)-resveratrol chelates represent a paradigm shift in metal-based cancer therapeutics, moving beyond nonspecific cytotoxicity to mechanism-driven, tumor-selective molecular targeting.

## Resveratrol co-delivered with the covalent organic framework (COF)

### Advantages of COF platforms

The integration of resveratrol (RSV), a naturally occurring polyphenolic compound, with covalent organic frameworks (COFs) represents a cutting-edge strategy for targeted cancer therapy. COFs have emerged as highly versatile drug delivery platforms due to their exceptional structural tunability, high porosity (>700 m²/g), and biocompatibility [128, 129]. These crystalline porous materials enable precise control over drug loading and release kinetics through tailored pore architectures and surface functionalities. For instance, fluorinated COFs such as DF-TAPB-COF exhibit remarkable loading capacities (e.g., 69% for 5-fluorouracil) and pH-responsive release profiles in tumor microenvironments (TME), leveraging the acidic pH (5.7–6.8) to trigger drug liberation via protonation of framework components. This pH sensitivity arises from the dynamic equilibrium between COF-drug interactions and TME-specific chemical conditions, ensuring minimal off-target toxicity while maximizing therapeutic efficacy [130, 131]. Despite this promising mechanism, the pH-responsive property presents a practical challenge: it may lead to premature drug leakage during systemic circulation if not meticulously controlled.

The pharmacological potency of RSV in cancer therapy stems from its multi-targeted mechanisms. As a chemosensitizer, RSV enhances the efficacy of conventional chemotherapeutics like 5-FU by modulating macrophage cytokine profiles and suppressing anti-apoptotic proteins such as Bcl-xL and Mcl-1 [132, 133]. Recent studies reveal its capacity to induce ferroptosis in colorectal cancer cells through ROS-dependent lipid peroxidation and downregulation of SLC7A11/GPX4 pathways, while simultaneously activating autophagy-related apoptosis via FOXQ1 inhibition [134–136]. Collectively, these dual mechanisms exploit metabolic vulnerabilities in cancer cells, particularly under the hypoxic and acidic conditions prevalent in solid tumors. When co-delivered with COFs, RSV benefits from the nanocarrier’s protective effects against rapid hepatic metabolism and systemic clearance, significantly improving its bioavailability and tumor accumulation [137, 138].

Structural optimization of COF platforms further enhances their therapeutic performance. DF-TAPB-COF demonstrates superior drug retention through fluorine-mediated hydrogen bonding with RSV, while its hierarchical porosity (2.3 nm average pore width) accommodates both hydrophobic drug molecules and hydrophilic targeting ligands. Comparative studies show fluorinated COFs achieve 2.2-fold higher drug loading than non-fluorinated analogs, attributed to enhanced van der Waals interactions and F–H bonding. The framework’s crystallinity and chemical stability, confirmed by PXRD and FT-IR analyses, ensure structural integrity during systemic circulation, while TGA data validate controlled thermal decomposition profiles for sustained release applications [139]. However, the reliance on fluorine for enhanced binding may raise concerns regarding potential fluorine-associated cytotoxicity or environmental persistence, which warrants further investigation (Table 4).

**Table 4:** Covalent organic frameworks (COFs) for resveratrol delivery

COF type	Functionalization	Drug loading capacity	Release trigger	Key advantage	Reference
DF-TAPB-COF	Fluorinated	69% (5-FU)	pH (5.0)	High porosity; controlled release	[129]
APTES-COF-1	PEG-curcumin	N/A	N/A	Enhanced stability; blue shift in PL	[144]
TP-Por COF	IR783/cisplatin	N/A	NIR	Photothermal + chemotherapy synergy	[147]

Clinical translation of this co-delivery system faces challenges in scalability and biocompatibility validation. Although in vitro cytotoxicity assays demonstrate >80% cell viability for DF-TAPB-COF at therapeutic concentrations, long-term biodistribution studies are needed to address potential immunogenicity. Recent advances in COF functionalization, including PEGylation and surface charge modulation, show promise in improving circulatory half-life and tumor-specific accumulation through EPR effects [140]. Parallel developments in stimuli-responsive nanocarriers highlight the importance of integrating multiple therapeutic modalities—RSV’s anti-angiogenic properties and COF-mediated photothermal effects could synergize for combinatorial therapy, as demonstrated in hybrid systems achieving 92% tumor growth inhibition in murine models [141].

Future research should prioritize structure-activity relationship studies to optimize COF-RSV interactions, alongside clinical trials evaluating pharmacokinetic profiles in heterogeneous tumor environments. The integration of machine learning for predictive drug release modeling and advanced imaging modalities for real-time therapeutic monitoring could accelerate the development of precision oncology platforms [142, 143]. As both COF chemistry and RSV’s molecular targets continue to be elucidated, this co-delivery paradigm exemplifies the convergence of nanomaterials engineering and phytochemical pharmacology in next-generation cancer therapeutics.

### Functionalized COFs

Resveratrol, a naturally occurring polyphenol with demonstrated chemopreventive and anti-inflammatory properties, has faced persistent challenges in therapeutic applications due to its poor aqueous solubility, rapid metabolism, and low systemic bioavailability [144]. Recent advancements in nanodelivery systems, particularly those involving covalent organic frameworks (COFs), have emerged as transformative solutions to these limitations. Among these innovations, functionalized COFs such as PEG-curcumin@COF nanocomposites represent a paradigm shift in dual-drug delivery strategies. These systems leverage the synergistic interactions between resveratrol and curcumin while addressing critical barriers in targeted delivery through structural and functional engineering of COF platforms [112]. The integration of polyethylene glycol (PEG) chains into COF architectures not only enhances colloidal stability and aqueous dispersibility but also modulates cellular uptake kinetics by reducing nonspecific protein adsorption and prolonging systemic circulation [145]. For instance, PEG2000-CCM@APTES-COF-1 nanocomposites exhibited superior water stability and a 75 nm blue shift in photoluminescence spectra, indicative of strong π-π stacking interactions between curcumin and the COF matrix that prevent premature drug leakage. While these functionalization strategies mark significant progress, it is critical to assess whether the enhanced stability and loading efficiency sufficiently address the inherent complexity of in vivo environments. The reliance on π-π stacking, although effective for encapsulation, may not universally apply to all therapeutic agents, particularly those lacking aromatic structures.

The structural superiority of COFs over conventional nanocarriers lies in their crystalline porosity, which enables precise drug loading through host-guest interactions. Powder X-ray diffraction (PXRD) analyses of APTES-COF-1 revealed maintained crystallinity post-functionalization, with distinct (110), (201), and (002) diffraction planes confirming structural integrity during drug encapsulation [145]. Thermogravimetric analysis (TGA) further demonstrated that PEG-CCM@APTES-COF-1 composites retain thermal stability up to 200 °C, a critical feature for maintaining drug integrity during storage and administration [146]. Comparative studies with traditional liposomal systems highlight COFs’ advantages: while liposomes suffer from burst release and lipid peroxidation, COF-based carriers achieve sustained release profiles over 4–6 days due to their robust covalent networks and reduced susceptibility to physiological degradation [147]. This extended release kinetics aligns with clinical needs for chronic disease management, particularly in cancer therapy, where prolonged drug exposure enhances therapeutic efficacy.

Functionalization strategies have also addressed the historical challenge of off-target effects in nanomedicine. The PEG corona in these nanocomposites reduces nonspecific cellular uptake by normal tissues through steric hindrance and surface charge modulation, as evidenced by flow cytometry showing 2.3-fold higher tumor cell internalization compared to non-targeted counterparts. Furthermore, folate receptor-mediated endocytosis can be integrated into COF designs, leveraging the overexpression of folate receptors in cancer cells to enhance specificity. Such targeting mechanisms, combined with pH-responsive drug release in tumor microenvironments (demonstrated by 80% cumulative 5-FU release from DF-TAPB-COF at pH 5.0), create spatially controlled delivery systems. These advancements are particularly relevant given that resveratrol’s systemic bioavailability remains below 5% in humans due to extensive first-pass metabolism, as shown in pharmacokinetic studies using 14C-labeled resveratrol [148].

Recent breakthroughs (2023-2024) in COF engineering have expanded their therapeutic scope through multimodal functionalities. The development of CaCO3@COF-BODIPY-2I@GAG nanostructures exemplifies this trend, combining calcium ion modulation with photodynamic therapy for enhanced antitumor effects. Similarly, TP-Por COF nanosheets loaded with IR783 and cisplatin derivatives demonstrate concurrent photothermal and chemotherapeutic activity, achieving 92% tumor growth inhibition in murine models [148]. These innovations address the critical need for combination therapies in overcoming multidrug resistance while maintaining biocompatibility—a key advantage of COFs over metal-containing frameworks like MOFs. Looking forward, the integration of artificial intelligence in COF synthesis optimization and the exploration of CRISPR-Cas9 co-delivery systems for gene-editing applications represent promising frontiers in personalized medicine [149]. However, challenges persist in scaling up production and fully understanding long-term biodistribution profiles, necessitating continued interdisciplinary collaboration between materials science and translational medicine [150].

## Discussion

The advancements in resveratrol (RSV)-based co-delivery systems outlined in this review underscore their transformative potential in addressing the persistent challenges of conventional cancer therapies. By leveraging biomaterial engineering, these systems synergistically enhance RSV’s multifaceted antitumor mechanisms—ranging from apoptosis induction and angiogenesis suppression to immunomodulation—while mitigating its pharmacokinetic limitations. The integration of RSV with chemotherapeutics, photothermal agents, and metal complexes has demonstrated remarkable tumor-specific cytotoxicity, as exemplified by pH-responsive liposomes and folate receptor-targeted nanoparticles that improve drug stability and bioavailability. Notably, stimuli-responsive platforms enable spatiotemporal control over drug release, optimizing therapeutic efficacy within the tumor microenvironment. However, the clinical translation of these systems remains hindered by heterogeneous tumor biology, inconsistent pharmacokinetic outcomes, and insufficient long-term biosafety data. For instance, while preclinical models show promising results with Pt(IV)-RSV prodrugs and Cu(II)-RSV chelates, their metabolic stability and organ-specific accumulation require further validation in human trials.

A critical gap lies in the incomplete understanding of structure-activity relationships (SARs) and the paradoxical effects of RSV’s pro-oxidant and antioxidant properties within co-formulations. Although RSV demonstrates potent antioxidant activity, its auto-oxidation under light exposure and rapid metabolism into sulfates/glucuronides significantly reduce its bioavailability and introduce unpredictable redox dynamics in vivo [151, 152]. Moreover, the concentration-dependent dual role of RSV—acting as an antioxidant at low doses but exacerbating oxidative stress at higher concentrations—adds complexity to its therapeutic window and necessitates precise dosing strategies in co-delivery systems to avoid unintended cytotoxic effects [153].

Current studies predominantly focus on optimizing nanocarrier designs—such as covalent organic frameworks (COFs) and graphene oxide hybrids—to achieve high drug-loading capacities and tumor-targeted delivery. Yet, challenges persist in balancing specificity and off-target effects, as evidenced by hepatic sequestration of folate-conjugated nanoparticles due to nonspecific uptake by Kupffer cells, sinusoidal endothelial cells, and hepatocytes, which remains a major hurdle for systemic delivery [154]. Additionally, the immunogenicity of certain polymeric systems, including PEGylated nanoparticles, can trigger unintended immune responses or accelerate clearance, further complicating clinical applicability [155, 156].

The biocompatibility of advanced materials like MOFs is another concern; while Zr- or Fe-based MOFs show lower toxicity, those incorporating Cu or Mn ions exhibit higher cytotoxicity linked to oxidative stress and incomplete biodegradation [157, 158]. Moreover, the formation of a “protein corona” on nanocarriers alters their surface properties and biological identity, potentially leading to opsonization, immune activation, and divergent pharmacokinetics [159]. For metal-based complexes (e.g., Pt(IV), Cu(II)), thermodynamic stability varies significantly with ligand choice and pH, influencing both efficacy and metal ion leakage, which may cause off-target toxicity or catalytic unintended reactions [160, 161].

The dual role of ROS in tumor promotion and suppression adds complexity: while RSV co-delivery systems can harness ROS for apoptosis induction, excessive or poorly controlled ROS generation may exacerbate tumor progression or damage healthy tissues [162, 163]. Furthermore, pharmacokinetic heterogeneity across patient populations, driven by genetic polymorphisms in metabolic enzymes or variable tumor microenvironment conditions, necessitates personalized dosing strategies yet remains underexplored in clinical settings [164].

The development of multifunctional platforms integrating imaging, targeting, and stimuli-responsive elements represents a promising frontier. For example, MOF-based systems combining chemotherapy, photothermal ablation, and ROS scavenging highlight the potential for multimodal therapy, though scalability and long-term biocompatibility remain key concerns, particularly regarding metal ion accumulation and organ-specific toxicity.

Future research must prioritize interdisciplinary efforts to bridge preclinical success and clinical utility. This includes pharmacogenomic stratification to identify responsive patient subpopulations, AI-driven optimization of nanoparticle synthesis, and combinatorial regimens with immunotherapies to convert immunologically “cold” tumors into “hot” microenvironments. Additionally, the exploration of metabolite-mediated mechanisms—such as bioactive RSV sulfates/glucuronides—could unlock novel therapeutic avenues. Rigorously designed clinical trials with biomarker-driven endpoints are essential to validate efficacy thresholds and establish standardized dosing protocols. Rigorously designed clinical trials with biomarker-driven endpoints are essential to validate efficacy thresholds and establish standardized dosing protocols, particularly for addressing inter-individual variability in RSV metabolism and nanocarrier clearance [165].

By addressing these challenges, RSV-based co-delivery systems may ultimately redefine precision oncology, offering a paradigm shift toward safer, more effective cancer therapies that harmonize natural phytochemistry with cutting-edge nanotechnology (Table 5).

**Table 5:** Challenges and Future Directions for RSV Co-Delivery Systems

Challenge	Current Status	Proposed Solution(s)	References
Pharmacokinetic & Bioactivity	Low oral bioavailability; rapid metabolism; pro-/antioxidant duality	Nano-encapsulation; prodrug design; stimuli-responsive release systems for precise dosing	[151–153]
Nanocarrier Biosafety & Targeting	Immunogenicity; protein corona formation; hepatic sequestration; metal ion leakage	“Stealth” coatings; biodegradable materials; active targeting ligands	[154–161]
Tumor Heterogeneity & Resistance	Heterogeneous TME; variable NP penetration; MDR; paradoxical ROS effects	Multi-stimuli responsive carriers; MDR reversal agents; ROS-modulating combination therapies	[162, 163]
Manufacturing & Translation Gap	Lab-scale synthesis; poor scalability; batch inconsistency; insufficient chronic toxicity data	AI-driven nanomaterial design; continuous nanomanufacturing; rigorous chronic toxicity studies	[157, 158, 165]
Immune System Interplay	Unintended immunogenicity; immunosuppressive TME; difficulty in activating immunity	Co-delivery with immunomodulators; carriers to repolarize TAMs; non-immunogenic materials	[155, 156, 165]
Clinical-Translational Barriers	PK heterogeneity; discordant preclinical-clinical outcomes; lack of biomarkers	Pharmacogenomic stratification; biomarker-driven trials; adaptive and personalized dosing	[164, 165]
Controlled Release & Specificity	Off-target release; insufficient tumor accumulation; poor spatiotemporal control	Stimuli-responsive systems; dual-targeting strategies; real-time monitoring with theranostics	[151, 152, 162, 163]
